# Simvastatin Inhibits Activation of NADPH Oxidase/p38 MAPK Pathway and Enhances Expression of Antioxidant Protein in Parkinson Disease Models

**DOI:** 10.3389/fnmol.2018.00165

**Published:** 2018-05-22

**Authors:** Huichun Tong, Xiuping Zhang, Xingjun Meng, Lingli Lu, Dongmei Mai, Shaogang Qu

**Affiliations:** ^1^Clinical Medicine Research Center, Shunde Hospital, Southern Medical University, Foshan, China; ^2^Teaching Center of Experimental Medicine, School of Basic Medical Sciences, Southern Medical University, Guangzhou, China

**Keywords:** Parkinson disease, simvastatin, NADPH oxidase, oxidative stress, anti-oxidase

## Abstract

Evidence suggests that oxidative stress is involved in the pathogenesis of Parkinson disease (PD). Simvastatin has been suggested to protect against oxidative stress in several diseases. However, the molecular mechanisms by which simvastatin protects against neuropathology and oxidative damage in PD are poorly elucidated. In this study, we aimed to investigate the potential neuroprotective effects of simvastatin owing to its anti-oxidative properties in 6-hydroxydopamine (6-OHDA)-treated SH-SY5Y cells and mice. The results of 2′,7′-dichlorodihydrofluorescein diacetate (DCFH-DA) fluorescence and CCK-8 assay demonstrated that simvastatin reduced intracellular reactive oxygen species (ROS) levels and reversed apoptosis in 6-OHDA-treated SH-SY5Y cells. Mechanistic studies revealed that 6-OHDA-induced activation of nicotinamide adenine dinucleotide phosphate (NADPH) oxidase/p38 mitogen-activated protein kinase (MAPK) pathway was inhibited and nuclear factor-κB (NF-κB) nuclear transcription decreased in SH-SY5Y cells after simvastatin treatment. Enhanced expression levels of superoxide dismutase (SOD), heme oxygenase-1 (HO-1), peroxisome proliferator-activated receptor-γ coactivator-1α (PGC-1α) and glutamate-cysteine ligase modifier subunit (GCLM) were observed after simvastatin treatment in 6-OHDA-treated SH-SY5Y cells. *In vivo* studies revealed that administration of simvastatin by gavage decreased limb-use asymmetry and apomorphine-induced rotations in 6-OHDA-lesioned mice. Simvastatin increased dopaminergic neurons and reduced protein tyrosine nitration and gliosis in the midbrain of PD mice. An inhibitory effect on activation of the NADPH oxidase/p38 MAPK was observed, and increased antioxidant protein expression in the midbrain were seen in the simvastatin plus 6-OHDA group compared with the 6-OHDA-lesioned group. Taken together, these results demonstrate that simvastatin might inhibit the activation of NADPH oxidase/p38 MAPK pathway, enhance antioxidant protein expression and protect against oxidative stress, thereby providing a novel antioxidant mechanism that has therapeutic validity.

## Introduction

Parkinson disease (PD) is the second most common neurodegenerative disorder following Alzheimer disease (Obeso et al., 2010). PD is characterized by tremor, bradykinesia, rigidity and postural instability (Olanow and Tatton, 1999; Vila and Przedborski, 2004). The pathological hallmark of PD comprises a marked loss of dopaminergic neurons in the substantia nigra pars compacta (SNc; Granado et al., 2013) and the presence of insoluble protein inclusions called Lewy bodies (Dunning et al., 2012). The prevalence of PD increases with age, being more than 1% in people over age 65 years and increasing to about 4% in those over age 85 years (Bekris et al., 2010; Crosiers et al., 2011). Current clinical dopamine replacement interventions provide symptomatic relief, but only on a temporary basis (Schapira, 2007; Skibinski and Finkbeiner, 2011). Thus, alternative strategies must be developed to modify the course of this disease.

Although PD has been heavily researched in the last two decades, the precise etiology of the disease is still unknown. It is noteworthy that research in recent years has provided substantial evidence supporting the hypothesis that oxidative stress is one of the critical factors that induce the onset of PD (Greenamyre and Hastings, 2004; Jing et al., 2015). Reports have found that nicotinamide adenine dinucleotide phosphate (NADPH) oxidase can be activated, producing a large quantity of related reactive oxygen species (ROS) in the brain of a PD mouse model, which play an important role in PD pathogenesis (Qin et al., 2013; Wang et al., 2015). An impaired antioxidant system has been observed in PD, including decreased superoxide dismutase (SOD), glutathione peroxidase (GSH-Px) activity and heme oxygenase-1 (HO-1) expression (Khurana and Gajbhiye, 2013; Lu et al., 2015). In addition, numerous studies have reported that use of antioxidant drugs prevents dopaminergic neuron death and neurobiological and behavioral deficits in PD, by virtue of anti-oxidative actions (Drummond et al., 2017; Michel et al., 2017). These studies suggest that antioxidant compounds or drugs might serve as potential therapeutic agents in PD, in addition to the available protective drugs (Hwang, 2013).

Simvastatin, a lipophilic statin that easily crosses the blood–brain barrier, is an inhibitor of 3-hydroxy-3-methyl-glutaryl-coenzyme A (HMG-CoA) reductase and is used worldwide as a cholesterol-lowering drug (Kostis et al., 2012; McGuinness et al., 2016). In addition to therapeutic use in hyperlipidemia, recent studies suggest that statins modulate neurodegeneration-related signaling processes and may be beneficial in PD and AD (Fassbender et al., 2001). It has been shown that lower serum levels of total cholesterol are associated with a significantly decreased risk of PD (Hu et al., 2008; Gao et al., 2012; Lee et al., 2013). A previous meta-analysis supports the hypothesis that statin use reduces the individual’s risk of PD (Undela et al., 2013), but no such effect was found for long-term statin use (Wolozin et al., 2007). In experimental parkinsonian models, simvastatin provides robust neuroprotection against dopaminergic neurodegeneration by anti-inflammatory mechanisms, cannabinoid receptor 1 and NMDA receptor modulation (Ghosh et al., 2009; Xu et al., 2013; Mackovski et al., 2016). However, in a study among only 124 participants, Huang et al. delineated that people with low levels of LDL cholesterol are more likely to have PD than those with high LDL levels (Huang et al., 2007). However, these authors did not report whether their patients had low LDL levels prior to their diagnosis of PD, or whether their LDL levels decreased after this diagnosis. Furthermore, that study was vastly underpowered in the sense that fewer than 20 of the 124 PD patients were actually taking statins so the results cannot be viewed as reliable. Different treatment time of simvastatin may lead to different results. The neuroprotective effect of simvastatin in PD remains controversial and the related mechanisms remain to be elucidated.

The neurotoxin 6-hydroxydopamine (6-OHDA) is commonly used to generate experimental models of PD by specifically inducing apoptosis in dopaminergic cells (Przedborski and Ischiropoulos, 2005; da Costa et al., 2009; Tobón-Velasco et al., 2013), including human SH-SY5Y cell lines (Guo et al., 2005; Arena et al., 2013). In the present study, we tested the hypothesis that simvastatin has therapeutic effects in 6-OHDA-induced cellular and animal models of PD via enhancing anti-oxidative effects.

## Materials and Methods

### Animals and Treatments

We obtained 8- to 10-week-old male C57BL/6 mice, weighing between 25 g and 30 g, from Guangdong Medical Animal Laboratory (Foshan, China). The animals had free access to water and food, and they were housed under a 12:12 h light/dark cycle. Experiments were performed with age and weight-matched animals. Before the experiments, the animals were allowed to adapt to the environment for at least 5 days. All animal experiments were performed in accordance with the National Institutes of Health Guide for the Care and Use of Laboratory Animals (Eighth Edition, 2011). All procedures were approved by the Institutional Animal Care and Use Committee of Southern Medical University (Guangzhou, China).

Animals were randomly divided into four groups: saline-treated sham-operated controls, simvastatin-treated group, 6-OHDA-treated group and simvastatin plus 6-OHDA treated group. The 6-OHDA was always made fresh before the experiments. Saline with 0.2% ascorbic acid was used to dissolve the 6-OHDA. We then filtered the 6-OHDA solution with a 0.2 μm bacterial filter. Based on the known dosage for statins (80 mg/day) in an adult human patient with hypercholesterolemia, we treated mice with a dose of 1 mg/kg body weight/day simvastatin (Ghosh et al., 2009; Mackovski et al., 2016; Sigma–Aldrich, St. Louis, MO, USA). Mice were treated with simvastatin or saline via gavage 2 days before the first injection of 6-OHDA (Sigma–Aldrich), followed by continuous administration of simvastatin for 14 days. On day 15 of lesioning, the mice were euthanized and the midbrains were quickly dissected out for assaying; the remaining samples were stored at −80°C.

### Neuroblastoma SH-SY5Y Cell Culture and Treatments

Human neuroblastoma SH-SY5Y cells were obtained from the Cell Bank of Type Culture Collection of the Chinese Academy of Sciences (Shanghai, China). The cells were maintained in Gibco Dulbecco’s Modified Eagle’s medium (Catalog number: 11965092, Thermo Fisher Scientific, Waltham, MA, USA) supplemented with 10% FBS (Thermo Fisher Scientific), 100 U/mL penicillin and 100 μg/mL streptomycin (Beyotime, Shanghai, China). The medium was subsequently changed every 2–3 days and cells were equilibrated in humidified air containing 5% CO_2_ at 37°C. The cultured cells were treated with 100 μM 6-OHDA and/or 1 μM simvastatin at the same time, for different lengths of time, as described below.

### Cell Counting Kit-8 (CCK-8) Assay

A total 1 × 10^4^ SH-SY5Y cells were seeded per well in 96-well plates. After 18–24 h, cells were incubated with 1 μmol/L simvastatin and/or 100 μmol/L 6-OHDA for another 24 h. Cell viability was determined using 2-(2-methoxy-4-nitrophenyl)-3-(4-nitrophenyl)-5-(2, 4-disulfophenyl)-2H-tetrazolium monosodium salt (WST-8) and monosodium salt with a CCK-8 assay (Enjing Biotech Co., Ltd., Nanjing, China), according to the manufacturer’s instructions. The absorbance was determined at 450 nm using a multimode plate reader (PerkinElmer Inc., Hopkinton, MA, USA).

### Measurement of Intracellular Reactive Oxygen Species (ROS)

Levels of oxidative stress were determined by measuring intracellular ROS generation using a 2′,7′-dichlorodihydrofluorescein diacetate (DCFH-DA) fluorescence assay (Beyotime). Briefly, SH-SY5Y cells were seeded in 96-well plates at a density of 1 × 10^4^/well and incubated overnight. After treatment with 6-OHDA and/or simvastatin, cells were incubated in serum-free medium containing 10 μM DCFH-DA at 37°C for 6 h. Each well was gently washed three times with sterilized phosphate-buffered saline (PBS) to remove uncombined DCFH-DA. Images were acquired using laser scanning confocal microscopy (Zeiss, Germany), with the same settings for all samples in each experiment. Fluorescence intensity was calculated using Image-Pro Plus 6.0 software (Silver Spring, MD, USA).

### 6-OHDA-Induced Lesions

Mice were deeply anesthetized with 4% chloral hydrate (loss of corneal and toe pad reflexes), and fixed on a stereotaxic instrument (Shenzhen Kingward Technology, Shenzhen, China) in a flat position. Mice received unilateral injections of 6-OHDA (5 μg/μL) or sterile saline in 2 μL volumes (at an injection speed of 0.3 μL/min) into the right side of the SNc at the following coordinates, according to the mouse brain atlas of Paxinos and Franklin (2001): anteroposterior (AP), −3.0 mm; lateral (L), −1.3 mm; and dorsoventral (DV), −4.7 mm (Park et al., 2014, 2016). Mice were left on a warming plate until they woke up from the anesthesia and were then returned to their home cages until use. To avoid dehydration, lesioned mice received sterile saline (10 mL/kg i.p.) for 3 days. In addition, during the first week post-surgery, food pellets soaked in water were placed in a shallow vessel on the floor of the cage.

### Apomorphine-Induced Rotation Test

Mice were placed individually in a cylinder (diameter: 23 cm; height: 30 cm) and allowed to adapt to their environment for 5 min. They were subsequently injected i.p. with apomorphine (Cayman Chemical, Ann Arbor, MI, USA) at a dose of 0.5 mg/kg. After 10 min, rotations were recorded for 30 min. Quantitative analyses of complete (360°) left rotations were made off-line by an investigator blinded to the experimental conditions. Results were expressed as the number of rotations to the side contralateral to the lesion.

### Cylinder Test

Forelimb use during exploratory activity was assessed using a cylinder test, as in previous studies (Park et al., 2014, 2016). Two weeks after 6-OHDA infusion, each mouse was individually placed inside a transparent glass cylinder (diameter, 15 cm; height, 22 cm) in which they could move freely. For each animal, an observer blinded to the identity of the animals observed a total of 20 contacts executed with the right and/or left forepaw on the wall of the cylinder. Use of the impaired (right) forelimb was expressed as a percentage of the total number of supporting wall contacts.

### Protein Extraction

For total protein extraction from SH-SY5Y cells, the culture medium was discarded, cells were washed twice with ice-cold PBS and harvested. For total protein extraction from midbrain samples, tissues were homogenized with a glass homogenizer, and cells or tissue homogenate was then centrifuged at 13,000× *g* for 2 min. Radioimmunoprecipitation assay (RIPA) buffer (Beyotime: contains 50 mM Tris (pH 7.4), 150 mM NaCl, 1% Triton X-100, 1% sodium deoxycholate, 0.1% SDS, sodium orthovanadate, sodium fluoride, EDTA, leupeptin) containing 1 mM phenylmethanesulfonyl fluoride (PMSF) was added, and the mixture was then placed on ice for 30 min. After centrifugation at 14,000× *g* for 15 min, the supernatant (containing the total protein fraction) was collected.

For extraction of cytoplasmic and nuclear fractions from SH-SY5Y cells, we use a Nuclear and Cytoplasmic Protein Extraction Kit (Beyotime), according to the manufacturer’s instructions. Briefly, cells were collected and resuspended in cytoplasmic protein isolation solution A. After vortexing for 5 s, the tubes were incubated for 15 min on ice to promote lysis. Next, cytoplasmic protein isolation solution B was added and the cells were vortexed for 5 s. The homogenate was centrifuged at 15,000× *g* for 5 min at 4°C. The supernatant containing the cytosolic fraction was immediately frozen for further analysis. The pellet was resuspended in nuclear protein isolation solution, vortexed and centrifuged at 15,000× *g* for 10 min. The resulting supernatant was the nuclear protein fraction.

For membrane protein extraction from midbrain samples, we used a Membrane Protein Extraction Kit (BioVision Inc., Milpitas, CA, USA), according to the manufacturer’s instructions. Briefly, 4 mL of cell wash solution was added to the midbrain tissue, vortexed briefly, centrifuged and the wash solution discarded. Then 1 mL of permeabilization buffer was added to the tissue, which was homogenized and then incubated for 10 min at 4°C with constant mixing. The mixture was centrifuged at 16,000× *g* for 15 min at 4°C to pellet the permeabilized cells. The supernatant (containing the cytosolic proteins) was carefully removed; the pellet was resuspended in 1 mL of solubilization buffer and incubated 30 min at 4°C, with constant mixing. The suspension was then centrifuged at 16,000× *g* for 15 min at 4°C. The supernatant (containing the solubilized membrane proteins) was collected and stored at −80°C for future use.

Protein concentrations were measured using a BCA assay (Beyotime). Samples were diluted with protein loading buffer and heated at 95°C for 5 min prior to western blot analysis.

### Western Blot

Immunoblot analysis was performed to detect the protein expression levels. Briefly, proteins were separated by SDS–PAGE gel (12% acrylamide gel). After the transfer, membranes were blocked with 5% bovine serum albumin (BSA) for 1 h. The blots were incubated with the primary antibodies at 4°C overnight, as follows: p38 mitogen-activated protein kinase (MAPK) antibody (1:1000; Enjing Biotech Co., Ltd., Nanjing, China), phospho-p38 MAPK (Thr180) antibody (1:1000; Enjing Biotech Co., Ltd.), glutamate-cysteine ligase modifier subunit (GCLM) antibody (1:1000; Enjing Biotech Co., Ltd.), peroxisome proliferator-activated receptor-γ coactivator-1α (PGC-1α) antibody (1:1000; Enjing Biotech Co., Ltd.), cleaved caspase-3 (Asp175, 5A1E) antibody (1:1000; Cell Signaling Technology Inc., Danvers, MA, USA), HO-1 antibody (1:200; Santa Cruz Biotechnology Inc., Santa Cruz, CA, USA), inducible nitric oxide synthase (iNOS) antibody (1:200; Santa Cruz Biotechnology Inc.), Bcl-2 antibody (1:200; Enjing Biotech Co., Ltd.), Bax antibody (1:200; Enjing Biotech Co., Ltd.), p47 phox antibody (1:200; Santa Cruz Biotechnology, Inc.), gp91 phox antibody (1:200; Santa Cruz Biotechnology, Inc.), tyrosine hydroxylase (TH; F-11) antibody (1:200; Santa Cruz Biotechnology Inc.), integrin β-1 (N-20) antibody (1:200; Santa Cruz Biotechnology Inc.), nitrotyrosine (11C2) antibody (1:200; Santa Cruz Biotechnology Inc.), β-actin antibody (1:1000; Beyotime) and glyceraldehyde-3-phosphate dehydrogenase (GAPDH) antibody (1:1000; Beyotime) and histone H3 antibody (1:200; Enjing Biotech Co., Ltd.). Subsequently, the blots were washed and incubated with secondary antibody diluted in blocking buffer for 1 h at room temperature. Secondary antibody included: HRP-labeled goat anti-mouse IgG (H+L; Beyotime), HRP-labeled goat anti-rabbit IgG (H+L; Beyotime) and HRP-labeled donkey anti-goat IgG (H+L; Beyotime). Bands were visualized using an enhanced chemiluminescence kit (Beyotime). Band density values of total proteins were normalized to GAPDH or actin, and band density values of membrane proteins were normalized to integrin. Band density was measured using ImageJ software. Data were collected from at least three independent experiments.

### Immunohistochemistry Assay

Immunohistochemistry assay was performed, as in our previous study (Zhang et al., 2017). Briefly, brain tissue samples were embedded in optimum cutting temperature compound (Sakura Finetek, Torrance, CA, USA) and stored at −80°C. Samples sections were cut into 10-μm slices and antigen retrieval was performed using citrate buffer. Sections were treated with 3% hydrogen peroxide (Sangon Biotech Co., Ltd., Shanghai, China) in PBS for 10 min and then incubated in 5% BSA for 10 min. Sections were incubated overnight at 4°C with primary antibodies as follows: TH (F-11) antibody (1:50; Santa Cruz Biotechnology Inc.), nitrotyrosine (11C2) antibody (1:50; Santa Cruz Biotechnology Inc.), glial fibrillary acidic protein (GFAP) antibody (1:50; Santa Cruz Biotechnology Inc.) and ionized calcium-binding adapter molecule 1 (IBA-1) antibody (1:500; WAKO, Osaka, Japan). After washing 3 times with PBS for 5 min each, sections were incubated sequentially in HRP-conjugated goat anti-mouse and goat anti-rabbit secondary antibody (ZSGB-BIO, Beijing, China) for 2 h at 37°C. Sections were visualized with a 3,3-diaminobenzidine (DAB) peroxidase substrate kit (Boster, Wuhan, China). Integrated option density (IOD) was determined using an Image-Pro Plus 6.0 photogram analysis system (IPP 6.0; Media Cybernetics, Bethesda, MD, USA).

### Immunocytofluorescence

Immunocytofluorescence was performed as in our previous study, with some modifications (Tong et al., 2017). Culture supernatant was discarded, and cells were then washed three times with pre-chilled PBS. Cells were fixed in cold 4% paraformaldehyde in PBS for 20 min and then washed three times with cold PBS. Cell slides were blocked with 5% BSA containing 0.3% Triton X-100 in PBS for 30 min at room temperature. Cells were incubated with primary antibodies at 4°C overnight as follows: anti-p47 phox antibody (1:100; Santa Cruz Biotechnology Inc.) and anti-nuclear factor-κB (NF-κB) antibody (1:100; Santa Cruz Biotechnology Inc.). The slides were incubated with FITC-conjugated goat anti-mouse antibody (1:300; ZSGB-BIO), and then kept in a dark place at room temperature. Images were acquired using laser scanning confocal microscopy (Zeiss), with the same settings for all samples in each experiment.

### Determination of Intracellular Superoxide Dismutase (SOD) Activity

Total SOD activity of the midbrain samples was determined using a Total SOD Assay Kit with WST-8 (Beyotime), based on the protocols provided by the manufacturer (Chen et al., 2017).

### Statistical Analysis

All experimental results were expressed as mean ± standard error of the mean (SEM) and analyzed using IBM SPSS 20.0 software (IBM Corp., Armonk, NY, USA). Statistical significance of the data was determined using Dunnett’s T3 test or Fisher’s least significant difference (LSD) *post hoc* test based on one-way analysis of variance (ANOVA). Significance was considered as *P* < 0.05. Data were drawn from at least three independent experiments.

## Results

### Simvastatin Protected Against 6-OHDA-Induced Cytotoxicity in SH-SY5Y Cells

**Figure 1:**
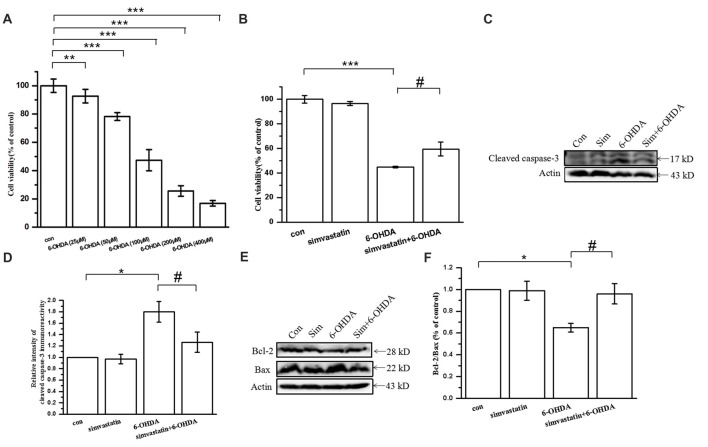
Simvastatin protected against 6-hydroxydopamine (6-OHDA)-induced cytotoxicity in SH-SY5Y cells. **(A)** SH-SY5Y cells treated with different concentration of 6-OHDA and cell viability determined after 24 h using a CCK-8 kit. **(B)** SH-SY5Y cells co-treated with 1 μmol/L simvastatin and 100 μmol/L 6-OHDA; cell viability determined after 24 h using a CCK-8 kit. **(C)** After 12 h co-treatment, expression of cleaved caspase-3 detected by western blot. **(D)** Relative quantitative analysis of cleaved caspase-3 expression. **(E)** Expression of Bcl-2 and Bax detected by western blot after 24 h co-treatment. **(F)** Analysis of the Bcl-2/Bax ratio. Data were obtained from three independent experiments. Statistical analysis was by Dunnett’s T3 test or least significant difference (LSD) *post hoc* test based on analysis of variance (ANOVA); data expressed as mean ± standard error of the mean (SEM). *P < 0.05, **P < 0.01, ***P < 0.001, ^#^*P* < 0.05.

To determine the effect of simvastatin on 6-OHDA-induced cytotoxicity, we exposed SH-SY5Y cells to simvastatin and/or 6-OHDA. The cell viability decreased to 16.8%–92.68% of control cells after treatment with 6-OHDA (25–400 μmol/L) for 24 h in SH-SY5Y cells (Figure 1A). Treatment with 100 μmol/L 6-OHDA significantly decreased the viability of SH-SY5Y cells after 24 h treatment (47.34 ± 7.40% of control cells). Next, 6-OHDA at 100 μmol/L was selected for the following experiments (Lin et al., 2007). Co-treatment with 1 μmol/L simvastatin plus 6-OHDA increased viability to 59.58 ± 5.80% (Figure 1B). After 12 h treatment, western blots showed that 100 μmol/L 6-OHDA also induced activation of caspase-3 (180.13 ± 10.44% of control cells; Figures 1C,D), which plays a key role in the terminal execution phase of apoptosis. Co-treatment with 1 μmol/L simvastatin plus 6-OHDA decreased cleaved caspase-3 expression to 126.46 ± 10.31%, compared with the 6-OHDA group. We further explored whether simvastatin changed the protein expression of Bcl-2 (anti-apoptotic) and Bax (pro-apoptotic) in 6-OHDA-treated SH-SY5Y cells. Co-treatment with 1 μmol/L simvastatin reversed the 6-OHDA-induced reduction in the Bcl-2/Bax ratio to 96.04 ± 9.29% after 24 h co-treatment (Figures 1E,F). These results suggest that simvastatin can effectively inhibit 6-OHDA-induced apoptosis in SH-SY5Y cells.

### Simvastatin Reduced 6-OHDA-Induced ROS and Inducible Nitric Oxide Synthase (iNOS) Production in SH-SY5Y Cells

**Figure 2:**
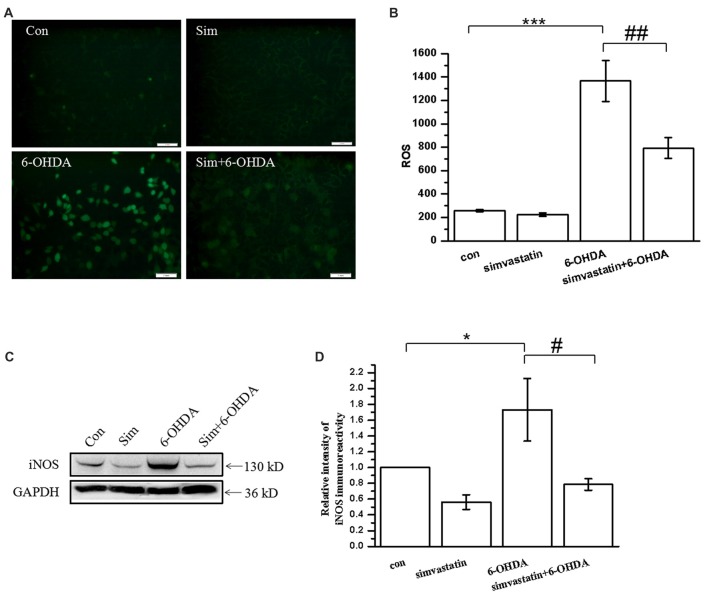
Simvastatin reduced 6-OHDA-induced reactive oxygen species (ROS) and inducible nitric oxide synthase (iNOS) production in SH-SY5Y cells. **(A)** SH-SY5Y cells treated with 1 μmol/L simvastatin and 100 μmol/L 6-OHDA for 6 h, and intracellular ROS was measured using the carboxy-H2DCFDA method. **(B)** Relative quantitative analysis of intracellular ROS. **(C)** SH-SY5Y cells treated with simvastatin and 6-OHDA for 24 h, and iNOS expression detected by western blot. **(D)** Relative quantitative analysis of iNOS expression. Scale bar, 50 μm. Data were obtained from three independent experiments. Statistical analysis was by Dunnett’s T3 or LSD *post hoc* test based on ANOVA; data are expressed as mean ± SEM. *P < 0.05, ***P < 0.001, ^#^*P* < 0.05, ^##^*P* < 0.01.

Oxidative stress, including by 6-OHDA, induces accumulation of a large quantity of ROS in SH-SY5Y cells (Lou et al., 2014). To examine whether simvastatin prevented the production of ROS after 6-OHDA treatment, the accumulation of ROS was measured in SH-SY5Y cells using the fluorescent probe DCFH-DA. As in a previous study, we found that 100 μmol/L 6-OHDA treatment alone induced an increase of the intracellular ROS level in SH-SY5Y cells after 6 h treatment (Figures 2A,B). However, co-treatment with 1 μmol/L simvastatin can inhibit 6-OHDA-induced production of ROS.

When the brain is exposed to oxidative stress, glial cells and neurons express a large quantity of iNOS, which results in sustained release of NO and leads to toxic effects (Bal-Price and Brown, 2001; Koppula et al., 2012). In this study, we found that 100 μmol/L 6-OHDA induced SH-SY5Y cells to produce large amounts of iNOS (173.13 ± 39.72% of control cells) after 24 h treatment (Figures 2C,D). Simvastatin significantly attenuated the expression of iNOS in 6-OHDA-treated SH-SY5Y cells (78.58 ± 7.32% of control cells).

### Simvastatin Can Inhibit 6-OHDA-Induced NADPH Oxidase Activation in SH-SY5Y Cells

**Figure 3:**
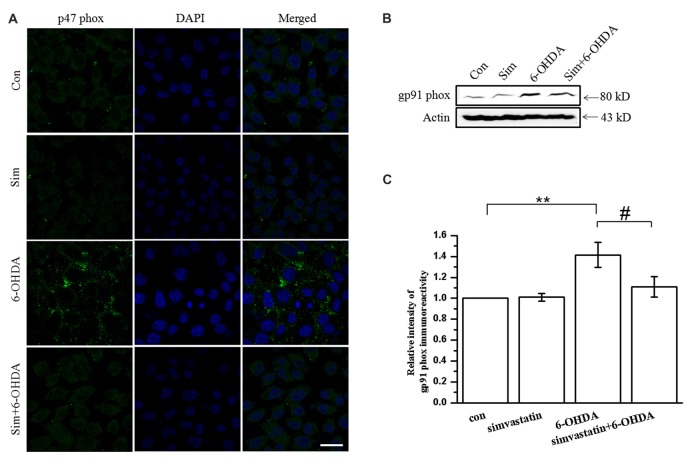
Simvastatin can inhibit 6-OHDA-induced nicotinamide adenine dinucleotide phosphate (NADPH) oxidase activation in SH-SY5Y cells. SH-SY5Y cells were co-treated with 1 μmol/L simvastatin or 100 μmol/L 6-OHDA. **(A)** Cell slides treated for 24 h and photographed after immunofluorescent staining, to detect levels of membrane p47 phox in SH-SY5Y cells. **(B)** Culture medium was discarded after 12 h treatment, total protein was extracted and total gp91 phox expression detected by western blot. **(C)** Relative quantitative analysis of gp91 phox expression. Scale bar, 30 μm. Data were obtained from three independent experiments. Statistical analysis by Dunnett’s T3 or LSD *post hoc* test based on ANOVA; data expressed as mean ± SEM. **P < 0.01, ^#^*P* < 0.05.

There are a variety of enzymes involved in the formation of ROS. NADPH oxidase is one of the most important sources of ROS production in the brain, and the translocation of cytosolic subunit p47 phox has a key role in the process of NADPH oxidase activation (Lambeth, 2004; Rastogi et al., 2017). We found that 100 μmol/L 6-OHDA promoted the translocation of p47 phox to the cell membrane in SH-SY5Y cells, as detected by immunofluorescence after 24 h treatment (Figure 3A), leading to the activation of NADPH oxidase. After treatment with 1 μmol/L simvastatin, the membrane translocation of 6-OHDA-mediated p47 phox was significantly attenuated, suggesting that the activity of NADPH oxidase was decreased. In addition, western blot showed that the expression of the gp91 phox subunit, another NADPH oxidase subunit, was significantly increased (141.38 ± 11.95% of control cells) after 6-OHDA treatment for 12 h, and co-treatment with simvastatin could reverse the process (110.99 ± 9.97% of control cells; Figures 3B,C).

### Simvastatin Can Inhibit 6-OHDA-Induced Activation of p38 MAPK and NF-κB Downstream of NADPH Oxidase in SH-SY5Y Cells

**Figure 4:**
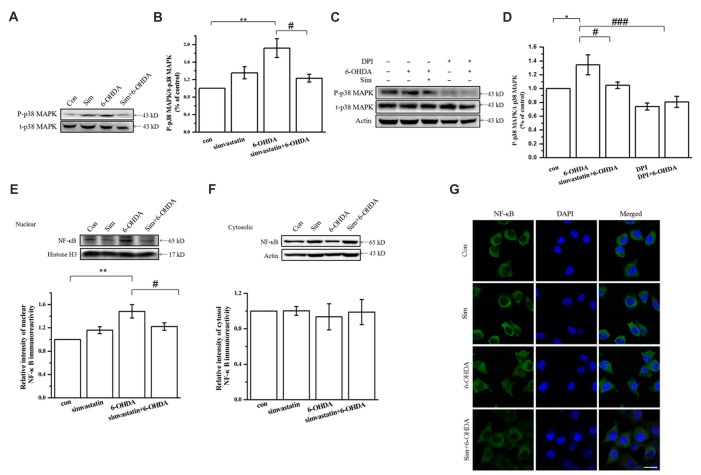
Simvastatin can inhibit 6-OHDA-induced activation of p38 mitogen-activated protein kinase (MAPK) and nuclear factor-κB (NF-κB) downstream of NADPH oxidase in SH-SY5Y cells. SH-SY5Y cells were co-treated with 1 μmol/L simvastatin and 100 μmol/L 6-OHDA. **(A)** Expression of Phospho-p38 mitogen-activated protein kinase (P-p38 MAPK) and t-p38 MAPK detected by western blot after 24 h. **(B)** Relative quantitative analysis of P-p38 MAPK and t-p38 MAPK expression. **(C)** After 5 μmol/L diphenyleneiodonium (DPI) pretreatment for 60 min, 1 μmol/L simvastatin and 100 μmol/L 6-OHDA were added to the culture; expression of P-p38 MAPK and t-p38 MAPK detected after 24 h. **(D)** Relative quantitative analysis of P-p38 MAPK and t-p38 MAPK expression in cells pretreated with DPI. Expression of nuclear NF-κB **(E)** and cytosolic NF-κB **(F)** detected by western blot after 6 h and relative quantitative analysis. **(G)** Cells were fixed, ruptured after 6 h co-treatment, then incubated with anti-NF-κB primary antibody and fluorescent-labeled secondary antibody. Nuclear translocation of NF-κB detected using confocal fluorescence microscopy. Scale bar, 30 μm. Data were obtained from three independent experiments. Statistical analysis by Dunnett’s T3 or LSD *post hoc* test based on ANOVA; data expressed as mean ± SEM. *P < 0.05, **P < 0.01, ^#^*P* < 0.05, ^###^*P* < 0.001.

NADPH oxidase activation induced by ROS and superoxide anions may cause p38 MAPK phosphorylation. As a common downstream target of inflammatory factors and oxidative stress, once activated, p38 MAPK can induce downstream signaling and further activate downstream NF-κB nuclear transcription, which mediates neuronal apoptosis. To define the role of simvastatin in the signaling molecule downstream of NADPH oxidase in SH-SY5Y cells after 6-OHDA treatment, we detected the activity of p38 MAPK and NF-κB. We found that Phospho-p38 mitogen-activated protein kinase (P-p38 MAPK) expression in SH-SY5Y cells was increased (134.39 ± 14.47% of control cells) after 100 μmol/L 6-OHDA treatment for 24 h; further, 1 μmol/L simvastatin could reverse 6-OHDA-induced activation of p38 MAPK (104.73 ± 4.77% of control cells; Figures 4A,B). Moreover, pretreatment for 1 h with 5 μmol/L diphenyleneiodonium (DPI), a potent NADPH oxidase inhibitor, also inhibited 6-OHDA-induced p38 phosphorylation activation in SH-SY5Y cells (Figures 4C,D), which is similar to the effect of simvastatin; this suggests a role of NADPH oxidase in mediating the activation of p38 MAPK. In addition, we found that 6-OHDA treatment for 6 h can activate NF-κB and promote its transcription to the nucleus. Immunoblot demonstrated that levels of nuclear NF-κB increased (148.29 ± 11.54% of control cells; Figure 4E), and 6-OHDA-mediated nuclear transcription can be inhibited (122.15 ± 6.47% of control cells) by simvastatin (Figure 4E). No significant difference was observed in cytosolic NF-κB among the different groups (Figure 4F). Similar results were found by immunocytofluorescence (Figure 4G).

### Simvastatin Exerted an Antioxidant Protective Effect by Enhancing Expression of Antioxidative Proteins in 6-OHDA-Treated SH-SY5Y Cells

**Figure 5:**
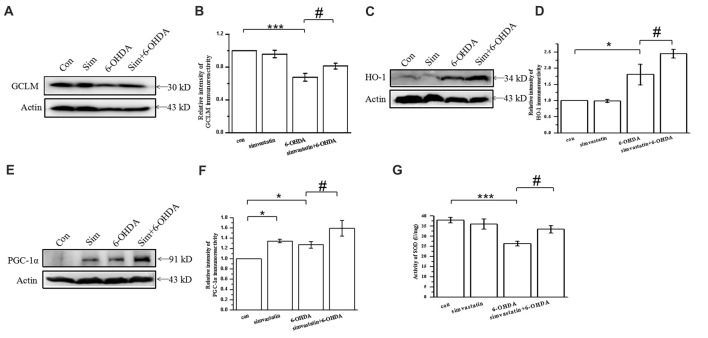
Simvastatin exerted an antioxidant protective effect by enhancing expression of antioxidative proteins in 6-OHDA-treated SH-SY5Y cells. SH-SY5Y cells exposed to 1 μmol/L simvastatin and 100 μmol/L 6-OHDA. **(A)** Expression of glutamate-cysteine ligase modifier subunit (GCLM) detected by western blot after 24 h. **(B)** Relative quantitative analysis of GCLM expression. **(C)** Expression of heme oxygenase-1 (HO-1) detected by western blot after 12 h. **(D)** Relative quantitative analysis of HO-1 expression. **(E)** Expression of peroxisome proliferator-activated receptor-γ coactivator-1α (PGC-1α) detected by western blot after 12 h. **(F)** Relative quantitative analysis of PGC-1α expression. **(G)** Total superoxide dismutase (SOD) activity of SH-SY5Y cells determined using a Beyotime Total SOD Assay Kit. Data were obtained from three independent experiments. Statistical analysis by Dunnett’s T3 or LSD *post hoc* test based on ANOVA; data expressed as mean ± SEM. *P < 0.05, ***P < 0.001, ^#^*P* < 0.05.

GCLM forms part of the rate-limiting enzyme complex in GSH synthesis and contributes to the effect against oxidase stress. Overexpression of GCLM may exert neurodegenerative effects in PD. In our study, we found that 6-OHDA decreased the expression of GCLM (67.49 ± 4.82% of control cells) in SH-SY5Y cells after 24 h treatment for 24 h (Figures 5A,B), which can be reversed by simvastatin plus 6-OHDA co-treatment (81.11 ± 3.55% of control cells).

It is widely accepted that stress-induced ROS can activate the antioxidant element to initiate expression of antioxidant genes encoding antioxidative proteins, including HO-1. Herein, we found that the expression of HO-1 in SH-SY5Y cells exposed to 100 μmol/L 6-OHDA for 12 h was unexpectedly increased (180.73 ± 32.11% of control cells; Figures 5C,D). Compared with the 6-OHDA group, the expression of HO-1 was further increased (245.02 ± 13.01% of control cells) after 1 μmol/L simvastatin plus 6-OHDA co-treatment, which exerted an antioxidant effect.

PGC-1α is a newly characterized transcriptional regulator that plays a key role in antioxidant stress systems. Elevated PGC-1α activity in neurons during oxidative stress can regulate cellular responses. To investigate the effect of simvastatin on PGC-1α, we examined the protein expression of PGC-1α in SH-SY5Y cells. Treatment with 1 μmol/L simvastatin or 100 μmol/L 6-OHDA alone increased the expression of PGC-1α to 134.43 ± 3.55% and 127.05 ± 6.44%, respectively, in SH-SY5Y cells (Figures 5E,F). Simultaneous administration of simvastatin and 6-OHDA could further enhance the expression of PGC-1α (159.18 ± 15.41% of control cells), compared with the 6-OHDA group.

In addition, 6-OHDA-induced reduction of SOD can be reversed by simvastatin (Figure 5G). Taken together, these data suggest that simvastatin up-regulates antioxidant response element (ARE) genes, leading to an increase in GCLM, HO-1, PGC-1α and SOD, which are potent cellular antioxidants.

### Simvastatin Improved Behavior Performance and Dopaminergic Neuronal Survival in the Unilaterally 6-OHDA-Lesioned Mouse Model of PD

**Figure 6:**
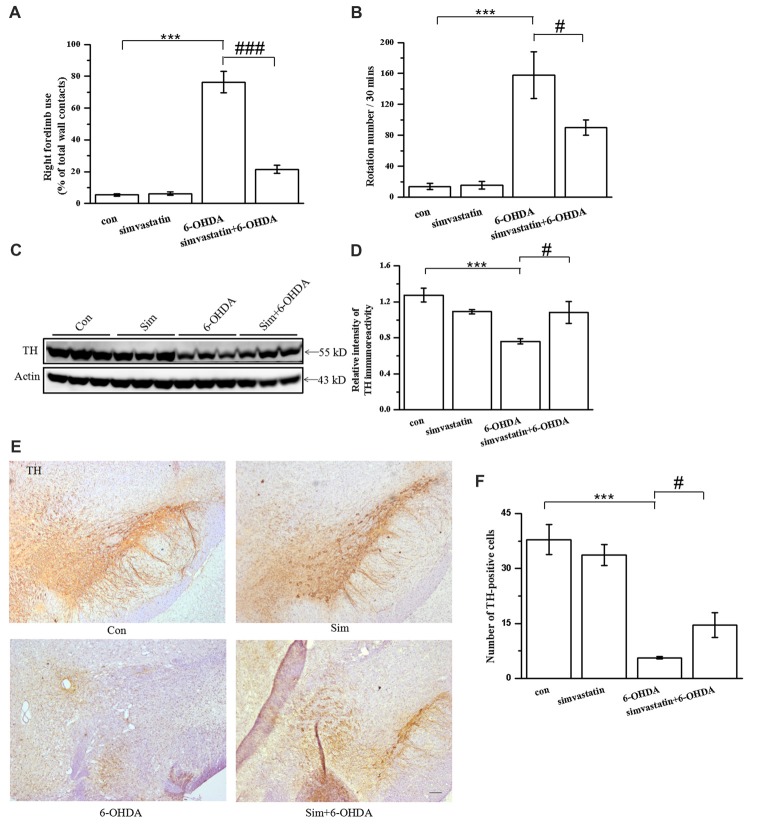
Simvastatin improved behavior performance and dopaminergic neuronal survival in the unilaterally 6-OHDA-lesioned mouse model of parkinson disease (PD). **(A)** Cylinder test was performed 2 weeks after 10 μg 6-OHDA stereotaxic injection of the substantia nigra pars compacta (SNc). A total of 20 contacts with the right or left forepaw on the wall of the cylinder were recorded; use of the right forelimb was expressed as a percentage of the total number of supporting wall contacts. Data were obtained from at least seven independent experiments. **(B)** Apomorphine-induced rotation 2 weeks after 6-OHDA stereotaxic injection was shown as the number of turns within 30 min. Data were obtained from at least five independent experiments. **(C)** Midbrain tissues were homogenized with radioimmunoprecipitation assay (RIPA) buffer. The supernatant contained the total protein after centrifugation; tyrosine hydroxylase (TH) expression was detected by western blot. **(D)** Relative quantitative analysis of TH expression. **(E)** Brain sections were prepared, incubated with TH antibody and HRP-conjugated secondary antibody, and visualized with 3,3-diaminobenzidine (DAB). **(F)** Relative quantitative analysis of TH-positive cells (dopaminergic neurons). TH-positive cells were counted using an IPP 6.0 system. Scale bar, 100 μm. Data were obtained from at least three independent experiments. Statistical analysis by Dunnett’s T3 or LSD *post hoc* test based on ANOVA; data expressed as mean ± SEM. ***P < 0.001, ^#^*P* < 0.05, ^###^*P* < 0.001.

To explore the effect of simvastatin on behavior performance in 6-OHDA-treated mice, we applied a cylinder test and apomorphine-induced rotation test after 2 weeks of 6-OHDA-induced lesioning. We found that the frequency of use of the right forelimb in 6-OHDA-lesioned mice was significantly higher (76.38 ± 6.83%) than that in controls (5.34 ± 0.85%), indicating that 6-OHDA-treated mice had limb-use asymmetry (Figure 6A). The use frequency of the right forelimb in 6-OHDA-treated mice was significantly reduced to 21.43 ± 2.61% after 1 mg/kg simvastatin treatment via gavage, indicating that simvastatin significantly improved the limb-use asymmetry. Moreover, 6-OHDA increased the number of apomorphine-induced rotations (6-OHDA: 157.80 ± 30.04; controls: 13.80 ± 3.89; Figure 6B). Mice treated with simvastatin plus 6-OHDA had a significantly decreased number of rotations (89.90 ± 9.96) compared with the 6-OHDA group, suggesting that simvastatin can attenuate 6-OHDA-induced dopamine neuronal injury in the lesioned side of the SNc. These data suggest that administration of simvastatin may alleviate the progress of behavior deficiency in a unilaterally 6-OHDA-lesioned mouse model of PD.

To explore the effect of simvastatin on TH-immunopositive neuronal cells in the midbrain, we used western blotting. TH-immunoreactivity in midbrain samples was greatly reduced in 6-OHDA-lesioned mice (6-OHDA: 0.76 ± 0.03; controls: 1.27 ± 0.08; Figures 6C,D). TH-immunoreactivity in the 6-OHDA-lesioned group increased to 1.08 ± 0.12 in the midbrain after administration of 1 mg/kg simvastatin via gavage. TH-immunopositive neurons in midbrain samples showed similar results using immunohistochemistry method (Figures 6E,F).

### Simvastatin Inhibited Gliosis and Level of Tyrosine Nitration in the Midbrain in the Unilaterally 6-OHDA-Lesioned Mouse Model of PD

**Figure 7:**
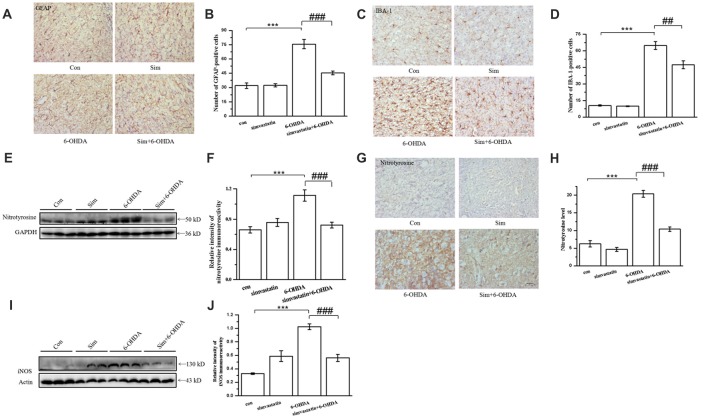
Simvastatin inhibited gliosis and the level of tyrosine nitration in midbrain samples in the unilaterally 6-OHDA-lesioned mouse model of PD. On day 15 after 6-OHDA stereotaxic injection, brain sections were prepared, incubated with glial fibrillary acidic protein (GFAP) antibody **(A)**, ionized calcium-binding adapter molecule 1 (IBA-1) antibody **(C)**, and HRP-conjugated secondary antibody, and then visualized with DAB. Relative quantitative analysis of GFAP-positive cells (astrocyte) **(B)** and IBA-1-positive cells (microglia) **(D)**. Data were obtained from at least three independent experiments. **(E)** Protein was extracted from midbrain tissues, and the level of nitrotyrosine was evaluated by western blot. **(F)** Relative quantitative analysis of nitrotyrosine expression. Data were obtained from four independent experiments. **(G)** Immunostaining of nitrotyrosine in the substantia nigra (SN); **(H)** immunoreactivity of nitrotyrosine was analyzed and quantified using IPP 6.0. Scale bar, 30 μm. **(I)** iNOS level evaluated by western blot. **(J)** Relative quantitative analysis of iNOS expression. Data were obtained from three independent experiments. Statistical analysis by Dunnett’s T3 or LSD *post hoc* test based on ANOVA; data expressed as mean ± SEM. ***P < 0.001, ^##^*P* < 0.01, ^###^*P* < 0.001.

It has been previously demonstrated that, in addition to the dramatic loss of dopaminergic neurons in the SNpc, gliosis is also a marked neuropathological feature in 6-OHDA-induced mouse models of PD (Wu et al., 2002). Herein, we found that the number of astrocytes (GFAP positive) in the substantia nigra (SN) of 6-OHDA-lesioned mice were dramatically increased (75.62 ± 4.78) in the lesioned side, in comparison with the control group (31.93 ± 2.97; Figures 7A,B). Similarly, the number of microglia (IBA-1 positive) from 6-OHDA-lesioned mice were significantly increased (64.80 ± 3.54). Administration of simvastatin decreased the number of astrocyte and microglia to 45.39 ± 1.88 and 47.40 ± 3.59, respectively, in the SN of 6-OHDA-lesioned mice (Figures 7C,D).

Previous studies have showed that nitration of TH occurred in mouse brain after 1-methyl-4-phenyl-1,2,3,6-tetrahydropyridine administration (Ara et al., 1998). Nitration of tyrosine residues in TH, a marker for protein nitration in the midbrain, results in loss of enzymatic activity, supporting a critical role for tyrosine nitration in TH inactivation. We further investigated the anti-oxidative effect of simvastatin on 6-OHDA-induced dopaminergic neuron degeneration by examining the level of tyrosine nitration. Using western blotting, we found that the level of tyrosine nitration in the midbrain of 6-OHDA-lesioned mice was greatly enhanced (densitometry: 1.11 ± 0.07) compared with the control group (densitometry: 0.66 ± 0.04; Figures 7E,F). Simvastatin administration inhibited elevated tyrosine nitration. This result was further confirmed using immunohistochemistry (Figures 7G,H). Moreover, increased expression of iNOS in SN after 6-OHDA lesioning was significantly inhibited by simvastatin (Figures 7I,J). These results suggest that the protective effects of simvastatin on 6-OHDA-induced neuronal degeneration may be mediated by its anti-oxidative effect.

### Simvastatin Inhibited the Activity of NADPH Oxidase and p38 MAPK in the Midbrain in the Unilaterally 6-OHDA-Lesioned Mouse Model of PD

**Figure 8:**
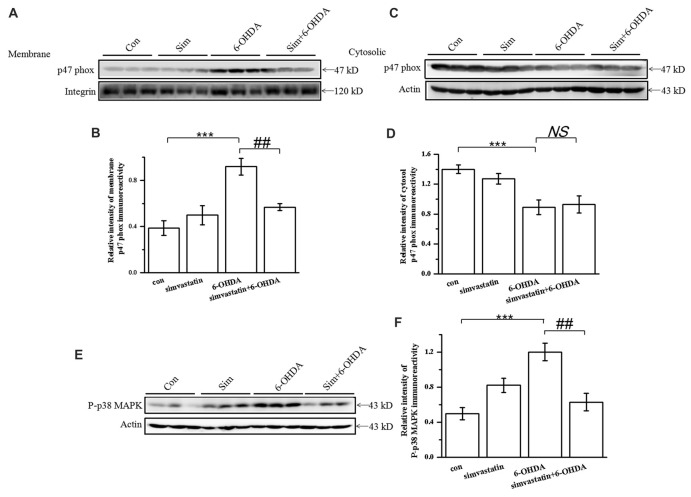
Simvastatin inhibited the activity of NADPH oxidase and p38 AMPK in midbrain samples in the unilaterally 6-OHDA-lesioned mouse model of PD. p47 phox subunit protein levels were determined in lysates of midbrain tissue. Supernatant containing solubilized membrane or cytosol proteins was collected; membrane p47 phox **(A)** and cytosol p47 phox **(C)** expression was detected using western blot. Relative quantitative analysis of membrane p47 phox **(B)** and cytosol p47 phox **(D)** expression performed using ImageJ. Data were obtained from three independent experiments. **(E)** Protein levels of P-p38 evaluated by western blot. **(F)** Relative quantitative analysis of P-p38. Data were obtained from five independent experiments. Statistical analysis by Dunnett’s T3 or LSD *post hoc* test based on ANOVA; data expressed as mean ± SEM. ***P < 0.001, ^##^*P* < 0.01. NS, not significant.

The expression of NADPH oxidase was up-regulated in the SN of the PD mouse model (Choi et al., 2012; Zhou et al., 2012). To define whether simvastatin exerted a protective effect by targeting NADPH oxidase in the midbrain, we detected the level and distribution of its subunit, p47 phox, in midbrain samples. We found that the level of membrane p47 phox was increased in 6-OHDA-lesioned mice (densitometry: 0.92 ± 0.07) compared with the control group (densitometry: 0.39 ± 0.06; Figures 8A,B), but the level of cytosol p47 phox was decreased (densitometry: 0.89 ± 0.10) compared with controls (densitometry: 1.40 ± 0.06; Figures 8C,D). After administration of simvastatin, the elevated level of membrane p47 phox in the midbrain was reversed (densitometry: 0.57 ± 0.03) in 6-OHDA-lesioned mice; however, there was no difference in the level of expression of p47 phox in the cytosol between the 6-OHDA group and simvastatin plus 6-OHDA group.

As p38 MAPK is correlated with NADPH oxidase activity, we continued to observe a change of p38 MAPK activity. After 6-OHDA stereotactic injection, the level of P-p38 MAPK in the midbrain increased to 0.82 ± 0.08 compared with that in the control group (densitometry: 0.50 ± 0.07; Figures 8E,F); The increased P-p38 MAPK induced by 6-OHDA was significantly attenuated (densitometry: 0.63 ± 0.10) by simvastatin administration. These data confirm that the therapeutic effect of simvastatin may also be related to the suppression of p38 MAPK activity.

### Simvastatin Enhanced the Expression of Anti-oxidative Signaling Molecules in the Midbrain in the Unilaterally 6-OHDA-Lesioned Mouse Model of PD

**Figure 9:**
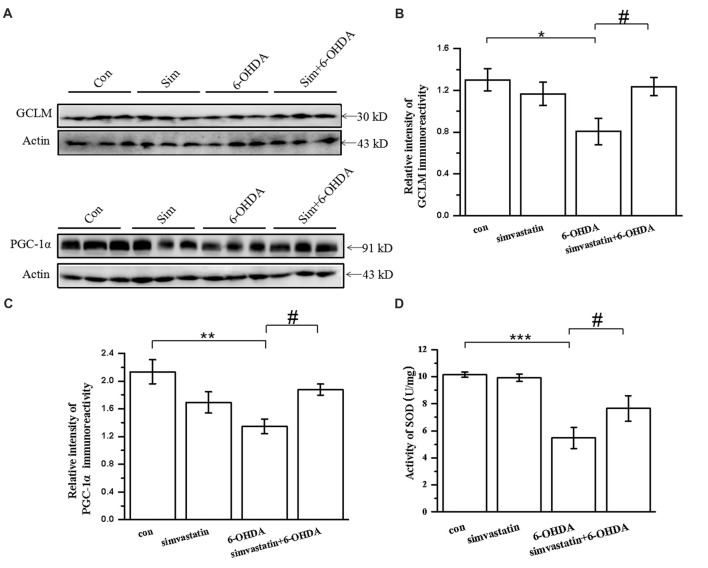
Simvastatin enhanced expression of anti-oxidative signaling molecules in midbrain samples in the unilaterally 6-OHDA-lesioned mouse model of PD. **(A)** Total protein in the midbrain was extracted with RIPA buffer, then western blot was used to detect the expression of GCLM and PGC-1α. Relative quantitative analysis of GCLM **(B)** and PGC-1α **(C)** expression performed using ImageJ. **(D)** Total SOD activity of midbrain samples determined using a Beyotime Total SOD Assay Kit; data were obtained from three independent experiments. Statistical analysis by Dunnett’s T3 or LSD *post hoc* test based on ANOVA; data expressed as mean ± SEM. *P < 0.05, **P < 0.01, ***P < 0.001, ^#^*P* < 0.05.

To investigate the change of oxidation resistance in 6-OHDA-treated mice after simvastatin treatment, we detected the anti-oxidative signaling pathway, including the expression of GCLM, PGC-1α and SOD. Oxidation resistance was decreased in 6-OHDA-treated mice, evidenced by reduced expression of GCLM (densitometry of 6-OHDA group: 0.81 ± 0.13; densitometry of controls: 1.30 ± 0.11) and PGC-1α (densitometry of 6-OHDA group: 1.35 ± 0.11; densitometry of controls: 2.13 ± 0.17; Figures 9A–C) in the midbrain. Simvastatin could reverse the reduction of GCLM (densitometry: 1.24 ± 0.09) and PGC-1α (densitometry: 1.88 ± 0.08) expression, which is consistent with the results in SH-SY5Y cells. Moreover, simvastatin could reverse 6-OHDA-induced reduction of SOD activity in the midbrain of PD mice (densitometry of 6-OHDA group: 5.47 ± 0.79; densitometry of simvastatin plus 6-OHDA group: 7.65 ± 0.93; Figure 9D).

## Discussion

PD is a neurodegenerative disorder involving the progressive degeneration of dopamine neurons in the SN (Olanow and Tatton, 1999). Although the etiology of PD has been extensively investigated for several decades, genetic and epigenetic factors remain elusive (Miller et al., 2009). Among all the pathological factors, oxidative stress is considered the predominant underlying mechanism for the progression of PD (Mythri et al., 2011). Therefore, use of antioxidants is considered a promising approach to slow the progression and limit the extent of neuronal cell loss in PD.

In this study, we demonstrated that simvastatin protected against 6-OHDA-induced cytotoxicity in dopaminergic neurons and reduced the intracellular ROS level in SH-SY5Y cells. In PD mice, simvastatin prevented protein tyrosine nitration and gliosis in addition to ameliorating behavior deficits and TH protein levels in the midbrain. Simvastatin inhibited 6-OHDA-induced NADPH oxidase activation by decreasing the translocation of p47 phox and expression of gp91 phox. In addition, simvastatin exerted an anti-oxidative effect by enhancing the expression of related antioxidant molecules including SOD, HO-1, PGC-1α and GCLM. Moreover, inhibition of p38 MAPK and NF-κB may be involved in the protective effects of simvastatin. Therefore, the protective effects of simvastatin against neuronal cell death in PD might be due to its anti-oxidative properties.

A recent study demonstrated that simvastatin is associated with decreased risk of PD (Brakedal et al., 2017). Simvastatin can also regulate the progression in PD models (Selley, 2005; Ghosh et al., 2009; Mackovski et al., 2016), but the mechanism that exerts the protective effects remains unknown. Herein, we found that simvastatin can inhibit 6-OHDA-induced apoptosis in SH-SY5Y cells, consistent with a previous study (Yan et al., 2014). Simvastatin can also reverse the reduction of dopaminergic neurons and behavior deficits in PD mice (Ghosh et al., 2009).

Oxidative stress has been associated with an oxidant–antioxidant imbalance and is thought to underlie defects in energy metabolism and induce cellular degeneration (Cui et al., 2004). Dopaminergic neurons are more susceptible to oxidative damage because of auto-oxidation and enzymatic oxidation of dopamine (Hanrott et al., 2006). Evidence suggests that oxidative stress caused by excess ROS production is thought to be associated with the development of PD (Cui et al., 2004). In recent years, simvastatin’s anti-oxidative capacity has shown a protective effect in various oxidative stress-related diseases (Chang et al., 2017). Based on previous studies of the anti-oxidative effects exerted by simvastatin, we sought to study these effects in a PD model. In our study, we observed excess production of ROS and increased iNOS expression in 6-OHDA-induced PD model, as in a previous study (Cui et al., 2016), which can lead to dopaminergic neuron death in the brain in PD. Simvastatin inhibited the production of ROS and overexpression of iNOS in PD model. Inhibition of glial activation might contribute to the protective effect of simvastatin in the PD model (Rodriguez-Pallares et al., 2007).

The presence of nitrotyrosine on proteins can be used as a marker for peroxynitrite formation *in vivo*. A previous study provided evidence that nitration at single or multiple tyrosine residues may be involved in the regulation of α-syn oligomerization and fibril formation (Burai et al., 2015), which may contribute to the pathogenesis of PD. We found that simvastatin can reverse the increased level of nitrotyrosine in the midbrain; however, further study is needed to verify whether increased nitrotyrosine is related to α-syn. In conclusion, consistent with our expectations, simvastatin exerted anti-oxidative effects in the PD model.

NADPH oxidase, a superoxide-producing enzyme, is composed of membrane (gp91 phox, p22 phox) and cytosolic (p47 phox, p67 phox, p40 phox and Rac1/2) subunits (Ansari and Scheff, 2011; Guemez-Gamboa et al., 2011; Khalyfa et al., 2014). Membrane translocation of cytosolic subunits is necessary for the activation of NADPH oxidase (Lambeth, 2004; Rastogi et al., 2017). Translocation of p47 phox to the plasma membrane may reflect the activation of NADPH oxidase activation (Hu et al., 2010; Wang et al., 2014; Hou et al., 2017b). Previous reports have found that NADPH oxidase can be activated and produce a large amount of related ROS in the brain of a PD model and that it plays a key role in chronic neuroinflammation and related dopaminergic neurodegeneration in PD (Peterson and Flood, 2012; Qin et al., 2013; Wang et al., 2015; Hou et al., 2017a). Therefore, the effects of simvastatin on NADPH oxidase were examined by measuring the membrane translocation of cytosolic subunits and level of gp91 expression in PD mice. In our study, we found increased membrane translocation of p47 phox and expression of gp91 phox in 6-OHDA-treated SH-SY5Y cells. We also found that the membrane level of gp91 phox in the midbrain of PD mice was elevated, as in previous studies (Rodriguez-Pallares et al., 2007; Jiang et al., 2015). After simvastatin treatment, the translocation of p47 phox and expression of gp91 phox was decreased in SH-SY5Y cells and PD mice, which suggests that simvastatin may down-regulate the activity of NADPH oxidase in the PD model, and reduce the formation of oxidative stress factor, to protect against the development of PD.

NADPH oxidase can activate extracellular signal-regulated kinase 1/2 (ERK1/2) and p38 MAPK along with the activation and translocation of NF-κB (Priya et al., 2017). p38 MAPK and NF-κB, two well-characterized oxidative stress-responsive pro-death signaling pathways, appear to participate in dopaminergic neuronal cell death in PD models (Tobón-Velasco et al., 2013). In our study, we observed the activation of p38 MAPK and NF-κB in PD models, and that simvastatin could effectively reserve this process. Moreover, addition of DPI exerted similar effects in SH-SY5Y cells, which suggests that the decrease of p38 MAPK and NF-κB activation may be partly caused by simvastatin-induced inhibition of NADPH oxidase activity.

An increasing number of studies have found that increased expression of anti-oxidase genes, including SOD (Filograna et al., 2016) and HO-1 (Song et al., 2017), is beneficial for the improvement of PD. Many studies have confirmed that simvastatin can inhibit epithelial–mesenchymal transition (Clark et al., 2016), reduce ventilator-induced lung injury (Zhao et al., 2015) and protect Neuro2a cells against lipopolysaccharide-induced damage by up-regulating anti-oxidase gene expression (Hsieh et al., 2011). Based on this evidence, we examined anti-oxidase gene expression in PD. Herein, we confirmed that simvastatin strongly enhanced the expression of stress proteins, including SOD, HO-1, PGC-1α and GCLM in the PD model, which may contribute to the removal of stress factor. Therefore, via enhancing its anti-oxidase capacity, simvastatin produced neuroprotective effects in PD.

Together, these findings demonstrate that simvastatin effectively exerts neuroprotective action in a 6-OHDA-induced PD model through the enhancement of antioxidant capability and attenuation of oxidative stress injury. Thus, simvastatin may offer a potential treatment to slow the progression of PD. However, further investigation in animals or humans is necessary to define the anti-oxidase capacity of simvastatin in PD. The results of this study also provide compelling evidence in the search for more potent pharmacological agents to protect against oxidative stress for treating neurodegenerative diseases, especially PD.

## Author Contributions

SQ planned and conducted all the experiments. HT, XZ, XM, LL and DM contributed to performing the experiments. HT contributed to performing the data analysis and drafting the manuscript. XZ revised the manuscript. All authors approved the final manuscript.

## Conflict of Interest Statement

The authors declare that the research was conducted in the absence of any commercial or financial relationships that could be construed as a potential conflict of interest.

## Abbreviations

6-OHDA: 6-hydroxydopamine
ANOVA: analysis of variance
ARE: antioxidant response element
DPI: diphenyleneiodonium
ERK1/2: extracellular signal-regulated kinases 1 and 2
GAPDH: glyceraldehyde-3-phosphate dehydrogenase
GCLM: glutamate-cysteine ligase modifier subunit
GSH-Px: glutathione peroxidase
HMG-CoA: 3-hydroxy-3-methylglutaryl-coenzyme A
HO-1: heme oxygenase-1
IBA-1: ionized calcium-binding adapter molecule 1
iNOS: inducible nitric oxide synthase
MAPK: mitogen-activated protein kinase
NADPH oxidase: nicotinamide adenine dinucleotide phosphate oxidase
NF-κB: nuclear factor-κB
PBS: phosphate-buffered saline
PD: Parkinson disease
PGC-1α: peroxisome proliferator-activated receptor-γ coactivator-1α
PMSF: phenylmethanesulfonyl fluoride
P-p38 MAPK: Phospho-p38 mitogen-activated protein kinase
ROS: reactive oxygen species
SEM: standard error of the mean
SN: substantia nigra
SNc: substantia nigra pars compacta
SOD: superoxide dismutase
TH: tyrosine hydroxylase
WST-8: 2-(2-methoxy-4-nitrophenyl)-3-(4-nitrophenyl)-5-(2,4-disulfophenyl)-2H-tetrazolium monosodium salt.
